# Supplementary material for: Women’s multidimensional empowerment index and essential newborn care practice in Bangladesh: The mediating role of skilled antenatal care follow-ups
Source: PLoS One. 2023 Feb 7;18(2):e0281369. doi: 10.1371/journal.pone.0281369 (PMC9904503; doi:10.1371/journal.pone.0281369)
Supplement: Attachment — Submitted filename: Response to Reviewers.docx [file pone.0281369.s001.docx]

**Authors’ Response to Journal Requirements and Reviewer’s Comments**

**Journal Requirements**

***Comment # 01***

Please ensure that your manuscript meets PLOS ONE's style requirements, including those for file naming.

***Authors’ Response:***

The PLOS ONE's style requirements have been followed in writing the manuscript.

***Comment # 02***

In the ethics statement in the Methods and online submission information, please ensure that you have specified (1) whether consent was informed and (2) what type you obtained (for instance, written or verbal, and if verbal, how it was documented and witnessed). If your study included minors, state whether you obtained consent from parents or guardians. If the need for consent was waived by the ethics committee, please include this information.

If you are reporting a retrospective study of medical records or archived samples, please ensure that you have discussed whether all data were fully anonymized before you accessed them and/or whether the IRB or ethics committee waived the requirement for informed consent. If patients provided informed written consent to have data from their medical records used in research, please include this information.

***Authors’ Response:***

It has been taken care of.

***Comment # 03***

Your ethics statement should only appear in the Methods section of your manuscript. If your ethics statement is written in any section besides the Methods, please move it to the Methods section and delete it from any other section. Please ensure that your ethics statement is included in your manuscript, as the ethics statement entered into the online submission form will not be published alongside your manuscript.

***Authors’ Response:***

The ethics statement has been moved to the Methods section in the revised manuscript.

**Reviewer Comments**

**Reviewer # 1**

Thank you for the opportunity to review this manuscript. The topic is important for public health issue, especially in maternal and child health. I have some comments to improve the manuscript:

Dear Reviewer:

Thank you very much for your effort in reviewing our manuscript and for your thoughtful suggestions. We have modified the manuscript according to your comment and detailed corrections are listed below, point by point. All changes are highlighted in blue color in this revised manuscript with track changes.

***Comment # 01***

Introduction: The topic of empowerment has to be introduced in this section. How is women empowerment defined? Is there a framework used to define women’s empowerment components? This must be justified and supported by literature.

***Authors’ Response:***

Thank you very much for your valuable comment. We have mentioned the women’s empowerment along with its dimensions in the introduction section of the revised manuscript (Please see the lines 91-100 of the revised manuscript file).

***Comment # 02***

Methods: Explain why author used weighting approach to create the empowerment component.

***Authors’ Response:***

The previous studies constructed multiple components or factors of women’s empowerment. But we constructed single composite score to define the level of women’s empowerment using a weighting approach. To construct the weighting score, we followed a study on multidimensional poverty measures proposed by Alkire and Foster (2011). The reason has been in the method section. (Please see the lines 203-207)

Reference. Alkire, S., & Foster, J. (2011). Counting and multidimensional poverty measurement. Journal of public economics, 95(7-8), 476-487.

***Comment # 03***

Results: In Table 2, column 4 is not clear.

***Authors’ Response:***

We revised the Table 2, and we included a footnote in the table for understanding the table.

***Comment # 04***

Discussion:

There could be more a more detailed comparison of the results here with previous results. As we go from the top quartile of empowerment to the bottom quartile, how large is the change in the probability of SANC or ENC in other studies? Even if the other studies use different methods and different empowerment measures, it should still be possible to the infer the differences between the top and bottom quartiles in these studies.

***Authors’ Response:***

Thank you very much for your comment. To the best of our knowledge, no research has been conducted on the relationship between women's empowerment and ENC practice. As a result, we were unable to compare our findings to those of other studies.

***Comment # 05***

Policy implications are not clear. Conclusions drawn are supported by findings.

***Authors’ Response:***

We revised the conclusion part in the revised manuscript.

**Reviewer # 2**

This is a useful and innovative study with potential policy implications. It is not certain to this reviewer whether the idea of using antenatal care-visits as a mediating factor between women’s empowerment and essential newborn care is completely new, but it is a refreshing approach, especially in measuring women’s empowerment from a multi-dimensional angle. However, there are several points, stated below, which need to be addressed satisfactorily before the manuscript may be considered for publication.

Dear Reviewer:

Thank you very much for your effort in reviewing our manuscript and for your valuable comments. We have modified the manuscript according to your comment and detailed corrections are listed below, point by point. All changes are highlighted in blue color in this revised manuscript with track changes.

***Comment # 01***

Language editing: The manuscript must be professionally edited for English, particularly for use of articles such as “the” and tense. The manuscript is supposed to describe and explain the findings of a current research, yet some of the sentences read as if they are reporting past research.

***Authors’ Response:***

Thank you very much. The manuscript has been revised accordingly.

***Comment # 02***

Writing style. The style of writing is often too cryptic, especially in the presentation of tables. Table and figure headings should be self-explanatory, and the source of all the tables and figures must be noted. Even if the tables and figures are drawn by the authors based on their analysis of data, these should be mentioned as such.

***Authors’ Response:***

It has been taken care of.

***Comment # 03***

Statistical analyses of data: In general, the idea of calculating the direct and indirect effects of women’s empowerment on ENC practice is noteworthy. However, please confirm whether it was necessary to adjust for complex design in conducting logistic regression on data extracted from BDHS 2017-18, which is based on complex sampling. Please also explain what was involved in “wrangling” the data, as BDHS 2017-18 data must have been cleaned before being released for use.

***Authors’ Response:***

Thank you very much for your valuable comments. BDHS data followed a complex survey design. In our revised manuscript, we revised our all analysis after addressing complex survey design using **svy** setting in Stata. We also took into account the cluster variation in our regression analysis using multilevel or mixed-effect binary logistic regression model. Again, the word “wrangling” was used in the statistical analysis section. Actually, in our selected dataset, some characteristics were missing for some individuals, and hence we discarded these individuals from our dataset. For this case, we used the mentioned word “wrangling”. In the revised manuscript, we delated the word.

***(Specific Comments)***

***Comment # 04***

Lines 14-15, and 138-139. Please explain why only home births are considered in this research. According to Bangladesh Demographic and Health Survey 2017-18 (BDHS 2017-18. Chapter 9), almost one half (49%) of the births in the preceding three years were delivered at a health facility.

***Authors’ Response:***

The Bangladesh demographic and health survey (BDHS), 2017-18 reported the essential newborn care practices for home births only, In the original data set, we also did not find the information on ENC indicators for institutional births. For this reason, we only included home births only in our study. We added this reason in the revised manuscript (Please see the lines: 152-154).

***Comment # 05***

Line 58. “intrapartum, conditions of labour”. Do you mean intrapartum conditions of labour (i.e. no comma)?

***Authors’ Response:***

We have written the words correctly on the manuscript.

***Comment # 06***

Lines 142-154. Re initial care for babies after birth: Does “cord care” include Used safe

delivery kit/bag or boiled blade during delivery, Applied chlorhexidine after umbilical

cord was cut and tied (BDHS 2017-18 report Table 9.24? Re joint groupings. Where have you shown in your manuscript the joint groupings for safe cord care and optimal thermal care respectively, reported from previous studies?

***Authors’ Response:***

We revised the lines 142-154. According to BDHS 2017-18 report cord care include used safe delivery kit/bag or boiled blade during delivery. The nothing applied to the cord or only chlorhexidine applied is another component of ENC.

***Comment # 07***

Lines 156-162. Re components of women’s empowerment index. Why is women’s education used as a barrier to accessing healthcare and not as a part of household decision making autonomy? How valid is the assumption to consider household assets as proxy for women’s assets, when in fact household assets are most likely to be held jointly with husbands and possibly members of the extended family?

***Authors’ Response:***

It has been taken care of. Women’s education is included in the social independence domain of women’s empowerment.

***Comment # 08***

Lines 162-166. What is the justification of assigning equal weights to each dimension? Why did you not use Factor Analysis and find the weights from Factor Loadings?

***Authors’ Response:***

The previous studies constructed multiple components or factors of women’s empowerment. But we constructed single composite score to define the level of women’s empowerment using a weighting approach. To construct the weighting score, we followed a study on multidimensional poverty measures proposed by Alkire and Foster (2011). The reason has been in the method section. (Please see the lines 203-207)

Reference: Alkire, S., & Foster, J. (2011). Counting and multidimensional poverty measurement. Journal of public economics, 95(7-8), 476-487.

***Comment # 09***

Lines 177-185. Formula for individual score. Please re-write the formula as it does not contain the symbol ‘n’ (noted in Line 180) and symbols denoting the weights of indicator and dimension.

***Authors’ Response:***

It has been corrected.

***Comment # 10***

Lines 191-196. Re the new WHO guidelines about at least eight Skilled ANC (SANC) visits. The new guidelines were formulated in 2016. In your analysis you have applied the new recommendations to births and pregnancies occurring in the three years preceding the survey, which was conducted in 2017-18, i.e. births and pregnancies occurring during 2015-2018 or before. As such, many births and associated SANC visits (or non-visits) must have happened before the new guidelines came into effect (when at least 4 SANC visits were recommended). Under such circumstances how justified are you in considering a cut-off of less than 8 and 8 or more SANC visits in analysing the mediating effects of skilled antenatal care?

***Authors’ Response:***

One of the objectives of this study is to examine whether ENC practice increases with the increase of ANC visits. For this reason, we have used the newly proposed cut off point for ANC visits to observe how it works on ENC practices. The reason has also been included in the revised manuscript.

***Comment # 11***

Line 198. “gender of index child”. It appears from an inspection of Table 2 that the index child is the child whose antenatal care is the subject of this study. As such, selecting “gender of the index child” as a control variable does not make sense because it is not possible to know the gender of the child at the antenatal stage, unless tests like ultrasound or amniocentesis are done, which most women would not have done in this case. Therefore, this variable should be removed from the list of control variables and the analysis should be re-done.

***Authors’ Response:***

We reanalyzed the data after discarding the variable “gender of index child”.

***Comment # 12***

Lines 207-211. Statistical Analysis. Please confirm whether it was necessary to adjust for complex design in conducting logistic regression on data extracted from BDHS 2017-18, which is based on complex sampling. Please also explain what was involved in “wrangling” the data, as BDHS 2017-18 data must have been cleaned before being released for use.

***Authors’ Response:***

BDHS data followed a complex survey design. In our revised manuscript, we revised our all analysis after addressing complex survey design using **svy** setting in Stata. We also took into account the cluster variation in our regression analysis using multilevel or mixed-effect binary logistic regression model. Again, the word “wrangling” was used in the statistical analysis section. Actually, in our selected dataset, some characteristics were missing for some individuals, and hence we discarded these individuals from our dataset. For this case, we used the mentioned word “wrangling”. In the revised manuscript, we delated the word.

***Comment # 13***

Table 2. Please make the table heading self-explanatory by stating the covariates of which indicator you have presented in the table based on what test. Please also provide, in parentheses the range of values of Low, Average and High Women’s Empowerment Index. What values are shown under the column heading “Good ENC Practice”? Are these the point estimates of each variable and their 95% Confidence Intervals? Please write the source of this table (and other tables and figures). If this table is based on your own calculations, then please write the source somewhat as this: “Computed by the authors based on their analysis of BDHS 2017-18 data”.

***Authors’ Response:***

It has been taken care of.

***Comment # 14***

Fig 1. (Should write in full, as “Figure 1”). Please calculate if possible, the Direct Effect of MEI on ENC Practice, and the effects of MEI on Skilled ANC and Skilled ANC on ENC Practice respectively and show them in the diagram.

***Authors’ Response:***

The Direct Effect of MEI on ENC Practice, and the effects of MEI on Skilled ANC and Skilled ANC on ENC practice were reported in Table 3.

***Comment # 15***

Fig 2(a).This figure (and its parent table in BDHS 2017-18-Table 9.24) appears a little confusing with respect to cord care and nothing applied to cord, both of which are indicators of cord care. Therefore, please explain how 86.7% of home delivered births in the last three years had Used safe delivery kit/bag or boiled blade during delivery, applied chlorhexidine after umbilical cord was cut and tied and 44.1% had nothing applied to cord. Should these two percentages not add to 100?

***Authors’ Response:***

According to BDHS 2017-18 report we measure the five ENC components. Cord care include used safe delivery kit/bag or boiled blade during delivery. The nothing applied to the cord or only chlorhexidine applied is another component of ENC. Therefore, these two percentages are not to be 100. It is noted that nothing applied to the cord means no substances are used after cutting the umbilical cord.

***Comment # 16***

Fig 2(b). “ENC practice is considered as a binary random variable, where 1 indicates good ENC and 0 for the poor ENC. A newborn is said to be received good ENC if all five components were practiced; whereas the practice is poor if he/she missed any of the components”. (Lines 151-164 of this manuscript). In the context of this definition, please explain the five categories of ENC practice displayed in Fig 2(b).

***Authors’ Response:***

It has been taken care of.

***Comment # 17***

Please also examine all the other diagrams (and tables) and check them for missing information or lack of clarity.

***Authors’ Response:***

It has been taken care of accordingly.

***Comment # 18***

Lines 410-422. In conclusion you have recommended “that a woman should be highly empowered …”. Can you suggest what specific measures should be taken to make women highly empowered in order to possess “not less than 75% of empowerment”?

***Authors’ Response:***

The conclusion has been revised accordingly.
