# Supplementary material for: Women’s multidimensional empowerment index and essential newborn care practice in Bangladesh: The mediating role of skilled antenatal care follow-ups
Source: PLoS One. 2023 Feb 7;18(2):e0281369. doi: 10.1371/journal.pone.0281369 (PMC9904503; doi:10.1371/journal.pone.0281369)
Supplement: Attachment — Submitted filename: Response to Reviewers.docx [file pone.0281369.s002.docx]

**Authors’ Response to Reviewer’s Comments**

**Reviewer # 2**

Dear Reviewer:

Thank you very much for your effort in reviewing our manuscript and for your valuable comments. We have modified the manuscript according to your comments, and detailed corrections are listed below, point by point. All changes are highlighted in blue color in this revised manuscript with track changes.

***Comment # 1***

Comment 10. “Lines 191-196.”Re the new WHO guidelines about at least eight Skilled ANC (SANC) visits. The new guidelines were formulated in 2016. In your analysis you have applied the new recommendations to births and pregnancies occurring in the three years preceding the survey, which was conducted in 2017-18, i.e. births and pregnancies occurring during 2015-2018 or before. As such, many births and associated SANC visits (or non-visits) must have happened before the new guidelines came into effect (when at least 4 SANC visits were recommended). Under such circumstances how justified are you in considering a cut-off of less than 8 and 8 or more SANC visits in analyzing the mediating effects of skilled antenatal care?” is not addressed satisfactorily (Lines 231-240 of the revised manuscript). Please explain the reason why you have considered a cut-off of less than 8 and 8 or more SANC visits while the previous guideline of 4 skilled ANC visits was still followed when most of the births included in your study took place.

***Authors’ Response:***

Thank you for your thoughtful observation. All pregnant women and adolescent girls should follow the updated WHO recommendation released in 2016 to receive antenatal care (ANC). The guideline is also intended for national and local policymakers, implementers and managers of national and local mother and child health programs, non-governmental and other organizations, public health policymakers and health professionals. In accordance with a human rights-based perspective, the guide strives to capture the complex nature of the concerns facing ANC health care practices and delivery, and to promote person-centered health and well-being over death and morbidity prevention. As a result, adherence to the guidelines is necessary. In this study, however, we also followed the WHO guideline on ANC visits (2016) to observe how eight or more ANC visits influence good essential newborn care practice. Despite the fact that many of the births and related ANC visits (or non-visits) included in the study occurred prior to the revised guidelines, our study will provide more evidence to follow the most recent WHO guideline on ANC visits to ensure appropriate ENC practice. The study looked into whether or not the new guideline works in terms of receiving good ENC before adopting it. Furthermore, the current WHO recommendation on ANC visits was used in the literature, which included data from the 2018 Demographic and Health Survey [1–3]. The reason has also been included in the revised manuscript. The changes are highlighted in the revised manuscript with blue color (See page- 13,14, lines- 228-235).

References

1.	Ahinkorah BO, Seidu AA, Budu E, Mohammed A, Adu C, Agbaglo E, et al. Factors associated with the number and timing of antenatal care visits among married women in Cameroon: Evidence from the 2018 Cameroon Demographic and Health Survey. J Biosoc Sci. 2021. doi:10.1017/S0021932021000079

2.	Imo CK. Influence of women’s decision-making autonomy on antenatal care utilisation and institutional delivery services in Nigeria: evidence from the Nigeria Demographic and Health Survey 2018. BMC Pregnancy Childbirth. 2022;22: 1–12. doi:10.1186/s12884-022-04478-5

3.	Tessema ZT, Tesema GA, Yazachew L. Individual-level and community-level factors associated with eight or more antenatal care contacts in sub-Saharan Africa: Evidence from 36 sub-Saharan African countries. BMJ Open. 2022;12: 1–8. doi:10.1136/bmjopen-2021-049379

***Comment # 2***

Comment 16. Your response “It has been taken care of”. How have you taken care of it? Have you removed Figure 2(b) and retained Figure 2(a) as the only Figure 2??

***Authors’ Response:***

Thank you very much for your comment. According to your previous comment, we updated the explanation of Fig. 2(b) in the revised manuscript. Both (a) and (b) are included in the Figure 2. The changes are highlighted in the revised manuscript with blue color (See page-15,16, lines-280-290).
